# Genome wide association study of thyroid hormone levels following challenge with porcine reproductive and respiratory syndrome virus

**DOI:** 10.3389/fgene.2023.1110463

**Published:** 2023-02-09

**Authors:** Angelica Van Goor, Alex Pasternak, Muhammed Walugembe, Nadya Chehab, Glenn Hamonic, Jack C. M. Dekkers, John C. S. Harding, Joan K. Lunney

**Affiliations:** ^1^ Animal Parasitic Diseases Laboratory, United States Department of Agriculture, Agricultural Research Services, Beltsville Agricultural Research Center, Beltsville, MD, United States; ^2^ Department of Animal Science, Purdue University, West Lafayette, IN, United States; ^3^ Department of Animal Science, Iowa State University, Ames, IA, United States; ^4^ Department of Large Animal Clinical Sciences, Western College of Veterinary Medicine, University of Saskatchewan, Saskatoon, SK, Canada

**Keywords:** porcine reproductive and respiratory syndrome, thyroid hormones, hypothyroidism, quantitative trait loci, growing pig, fetal pig, immunity, disease resistance

## Abstract

**Introduction:** Porcine reproductive and respiratory syndrome virus (PRRSV) causes respiratory disease in piglets and reproductive disease in sows. Piglet and fetal serum thyroid hormone (i.e., T3 and T4) levels decrease rapidly in response to Porcine reproductive and respiratory syndrome virus infection. However, the genetic control of T3 and T4 levels during infection is not completely understood. Our objective was to estimate genetic parameters and identify quantitative trait loci (QTL) for absolute T3 and/or T4 levels of piglets and fetuses challenged with Porcine reproductive and respiratory syndrome virus.

**Methods:** Sera from 5-week-old pigs (N = 1792) at 11 days post inoculation (DPI) with Porcine reproductive and respiratory syndrome virus were assayed for T3 levels (piglet_T3). Sera from fetuses (N = 1,267) at 12 or 21 days post maternal inoculation (DPMI) with Porcine reproductive and respiratory syndrome virus of sows (N = 145) in late gestation were assayed for T3 (fetal_T3) and T4 (fetal_T4) levels. Animals were genotyped using 60 K Illumina or 650 K Affymetrix single nucleotide polymorphism (SNP) panels. Heritabilities, phenotypic correlations, and genetic correlations were estimated using ASREML; genome wide association studies were performed for each trait separately using Julia for Whole-genome Analysis Software (JWAS).

**Results:** All three traits were low to moderately heritable (10%–16%). Phenotypic and genetic correlations of piglet_T3 levels with weight gain (0–42 DPI) were 0.26 ± 0.03 and 0.67 ± 0.14, respectively. Nine significant quantitative trait loci were identified for piglet_T3, on *Sus scrofa* chromosomes (SSC) 3, 4, 5, 6, 7, 14, 15, and 17, and collectively explaining 30% of the genetic variation (GV), with the largest quantitative trait loci identified on SSC5, explaining 15% of the genetic variation. Three significant quantitative trait loci were identified for fetal_T3 on SSC1 and SSC4, which collectively explained 10% of the genetic variation. Five significant quantitative trait loci were identified for fetal_T4 on SSC1, 6, 10, 13, and 15, which collectively explained 14% of the genetic variation. Several putative immune-related candidate genes were identified, including *CD247*, *IRF8*, and *MAPK8*.

**Discussion:** Thyroid hormone levels following Porcine reproductive and respiratory syndrome virus infection were heritable and had positive genetic correlations with growth rate. Multiple quantitative trait loci with moderate effects were identified for T3 and T4 levels during challenge with Porcine reproductive and respiratory syndrome virus and candidate genes were identified, including several immune-related genes. These results advance our understanding of growth effects of both piglet and fetal response to Porcine reproductive and respiratory syndrome virus infection, revealing factors associated with genomic control of host resilience.

## 1 Introduction

Porcine reproductive and respiratory syndrome virus (PRRSV) infection causes respiratory disease in swine of all ages and reproductive disease in pregnant females (Lunney et al., 2016). PRRSV is highly infectious, persistent, and exceptionally variable. PRRS results in significant economic losses, last estimated at $664 million dollars per year in the U.S. alone (Holtkamp et al., 2013). Although vaccination and biosecurity efforts have partially reduced losses associated with PRRS, more opportunities exist to incorporate favorable genetics and identify novel biomarkers for selection of resilience replacement stock. Previous works have established a strong host genetic component to piglet (Dekkers et al., 2017) and fetal (Harding et al., 2017) response to PRRSV challenge, indicating potential to improve host response to infection.

Thyroid hormones affect a wide range of biological processes, such as growth (Cabello and Wrutniak, 1989), metabolism (Mullur et al., 2014), and development (Forhead and Fowden, 2014). The thyroid gland produces two main hormones: triiodothyronine (T3) and thyroxin (T4). T3 is the more bioactive derivative, although T4 is more abundant. Under homeostatic conditions, these two hormones are under tight regulation by the hypothalamic-pituitary-thyroid axis (HPT). In swine, thyroid hormone levels and associated HPT axis products have been shown to be altered due to feed intake (Buonomo and Baile, 1991), diet (Spiegel et al., 1993; Carroll et al., 1998), lipopolysaccharide (LPS) challenge (Castro et al., 2013), and PRRSV challenge (Pasternak et al., 2020b; Pasternak et al., 2021). The link of thyroid hormones with innate immunity (Montesinos and Pellizas, 2019) and adaptive immunity (Rubingh et al., 2020) has been established. Severe illness can result in a dramatic decrease in serum T3 and T4 levels, collectively called non-thyroidal illness syndrome (NTIS) (Wajner and Maia, 2012). Previous research has shown that piglet and fetal serum thyroid hormone (i.e., T3 and/or T4) levels decrease rapidly in response to PRRSV infection (Pasternak et al., 2020b; Pasternak et al., 2021; Ko et al., 2022). Recent assessment of fetal thyroid hormone levels following late gestation PRRSV challenge demonstrated a significant relationship between hormone levels and previously identified single nucleotide polymorphisms (SNPs) associated with fetal survival (Ko et al., 2022). However, the genetic control of T3 and T4 levels during infection is not completely understood. There have been few studies on the genetic influence of thyroid hormone levels in food animals. A recent study in Holstein cattle estimated the heritability of T3 and T4 at 11% and 19%, respectively, and found quantitative trait loci (QTL) associated with non-disease challenge levels (Gan et al., 2020). To our knowledge, we are the first to report heritabilities, genetic correlations, and QTL for thyroid hormone levels in swine following PRRSV challenge. The ability to maintain homeostatic levels of T3 and T4 during infection is optimal for continued growth and development, especially of commercial pre- and post-natal pigs.

This study used samples from two of the largest studies conducted to date on host responses to PRRSV infection. This includes samples on respiratory PRRS using the nursery piglet challenge model from the PRRS Host Genetics Consortium (PHGC) (Dekkers et al., 2017). Groups of 200 piglets from multiple genetics suppliers were challenged with one of two strains of PRRSV type 2 (PRRSV2) at approximately 28 days of age and weight gain (WG) and viral load (VL) were assayed until 42 days post inoculation (DPI). Samples from reproductive PRRS were collected from the pregnant gilt model (PGM) challenge trials (Harding et al., 2017). Pregnant gilts were challenged with PRRSV2 at gestation day ∼85 and fetal samples were collected at 12 or 21 days post maternal inoculation (DPMI), depending on the experiment. Specifically, our objectives were to estimate genetic parameters, identify QTL, and investigate nearby candidate genes for T3 and/or T4 levels of piglets and fetuses challenged with PRRSV to elucidate the genomic influence associated with resilience to reproductive and respiratory PRRSV infection.

## 2 Materials and methods

### 2.1 Ethics

For the piglet trials, all experimental protocols were approved by the Kansas State University Institutional Animal Care and Use Committee. For the fetal samples, all experimental protocols were approved by the University of Saskatchewan’s Animal Research Ethics Board (Protocol # 20160023) and adhered to the Canadian Council on Animal Care Guide to the Care and Use of Experimental Animals. Animals were donated from commercial companies for the PHGC experiments. Pregnant gilts were purchased from Fast Genetics Inc., for the PGM experiments. This study is reported in accordance with ARRIVE guidelines.

### 2.2 Animal experiments and datasets

#### 2.2.1 Piglets

Detailed descriptions of the piglet trials conducted by the PHGC have been previously published (Lunney et al., 2011) and described more specifically for the trials used in the current study by Hess et al. (2016). Briefly, 1792 animals from 12 of the PHGC trials were used for this study. Piglets were commercially sourced from North American breeding companies. Pig genetics varied by trial, and all piglets came from farms that were affirmed to be free of PRRSV, *Mycoplasma hyponeumoniae*, and swine influenza. Animals were transported to Kansas State University at approximately 21 days of age, randomly placed into 10–15 pens, and acclimatized for 7 days. Piglets were inoculated intramuscularly and intranasally with either NVSL-97-7985 or KS-2006-72109 PRRSV2 isolates; all animals used in the current study were challenged and animal was considered the experimental unit. Authors were aware of group allocation at the different stages of the experiment. A subset of phenotypic and genotypic data collected as part of the PHGC trials and stored in the secure PHGC database was used in the present study. Specifically, to study WG, body weight (BW) was measured at 0, 21, and 42 DPI. Piglet WG from 0 to 21 DPI (WG21) and WG from 0 to 42 DPI (WG42) were included in the genetic parameter analyses. PRRSV2 RNA concentration tested from sera at 11 DPI and then log10 transformed for future analyses. Pigs were euthanized at 42 DPI except in Trials 7 and 8, where they were euthanized at 35 DPI due to facility availability limitations. Weights from animals in trial 9 were excluded from the dataset as the animals were from the Iowa State University Residual Feed Intake selection lines (Cai et al., 2008). Animals were humanely euthanized by pentobarbital overdose following the American Veterinary Medical Association guidelines for the euthanasia of animals, and all efforts were made to minimize suffering. Serum VL was measured using a semi-quantitative Taq-Man PCR for PRRSV2 RNA described previously (Boddicker et al., 2012). Ear tissues were collected from all pigs for DNA isolation. Trials 1-9 were genotyped on the Illumina Porcine SNP60 Beadchip version 1 (San Diego, CA) at GeneSeek Inc. (Lincoln, NE) while samples from trials 10–15 were genotyped on the Illumina Porcine SNP60 Beadchip version 2 (San Diego, CA) at Delta Genomics (Edmonton, Alberta).

#### 2.2.2 Fetuses

The experimental procedures used for the two PGM trials providing the fetal samples have been previously described in great detail (Ladinig et al., 2014c; Pasternak et al., 2020b). In PGM1, 114 pregnant gilts at 85 (±1) days of gestation were randomly chosen to be infected with NVSL97–7895 at the University of Saskatchewan (Ladinig et al., 2015); all animals used in the current study were challenged and fetus was considered the experimental unit. In PGM2, 31 gilts were similarly inoculated at 84 (±0.5) days of gestation (Pasternak et al., 2020b). All gilts in both PGM1 and PGM2 experiments were purebred Landrace bred to homospermic Yorkshire semen sourced from Fast Genetics Inc. (Spiritwood, Saskatchewan). Authors were aware of group allocation at the different stages of the experiment. Prior to infection all gilts were confirmed to be free of PRRSV infection (Ladinig et al., 2015). At 12 (PGM2) or 21 (PGM1) DPMI, gilts and fetuses were euthanized by intravenous barbiturate overdose (Euthanyl Forte, Bimeda MTC Animal Health, 16,200 mg/gilt, ∼75–80 mg/kg) and a total of 1,276 fetuses (combined PGM1 and 2) were collected. Fetal preservation status was determined based on external appearance and presence of blood and pulsations in the umbilical cord as: viable (white skin, pulsing blood in umbilical cord), meconium staining on the head only, or meconium staining on the body, decomposed (dead, largely normal skin color, minimal edema) or autolyzed (dead, discolored externally, edematous). Blood was collected from the axillary vessels of viable and meconium-stained fetuses but not from dead fetuses which were not included in the present study. Fetal BW, brain weight, and liver weight were measured, and the thymus dissected and snap frozen in liquid nitrogen. The brain:liver ratio of weights was calculated as an indicator of intrauterine growth restriction (IUGR). PRRSV2 RNA concentration was quantified in the fetal thymus using an in-house probe-based RT-qPCR specific for the inoculum strain as described previously (Ladinig et al., 2014c) and then log10 transformed for future analyses. A total of 818 PGM1 fetuses were genotyped on the Illumina Porcine SNP60 Beadchip version 2 (San Diego, CA) at Delta Genomics (Edmonton, Alberta), while 458 PGM2 fetuses were genotyped on the Affymetrix Axiom Porcine Genotyping Array 650 K Beadchip (Santa Clara, CA) at Delta Genomics.

#### 2.2.3 Thyroid hormone measurements

Our investigations focused on serum thyroid hormone levels at 11 DPI in piglets, as T3 hormone levels were most extremely suppressed at this timepoint post PRRSV challenge (Pasternak et al., 2021). Fetal T3 and T4 levels were measured on sera collected on day of termination at 12 or 21 DPMI, depending on the animal experiment the fetuses were derived from (Pasternak et al., 2020b). There are two primary reasons we have not used the T3:T4 ratio in our analysis. The first is that this value is typically evaluated in the human clinical setting, particularly when evaluating the response to levothyroxine treatment. The value in this measure is derived from an established ratio of production (1:13) in the healthy thyroid, however such a ratio has not been effectively established in a healthy fetal pig throughout gestation. The second stems from our past investigations into fetal thyroid hormone response to PRRSV (Pasternak et al., 2020b; Ko et al., 2022) which indicates that the relative response of these two hormones is dependent on fetal phenotype. In short fetuses classified as viable show a decrease in both T3 and T4 while those classified as meconium stained primarily have a decrease in T4 while maintaining near normal levels of T3. A genome-wide association study (GWAS) analysis on the ratio is therefore likely to identify SNPs associated with the phenotype rather than those directly associated with thyroid hormone. All sera was stored at −20°C or −80 °C, respectively, until used to test total T3 (piglet_T3, or fetal_T3, ng/dL) or T4 (fetal_T4, µg/dL) levels using commercial RIA kits (MP Biomedical, Irvine, CA) as previously described (Pasternak et al., 2020b).

#### 2.2.4 SNP analyses

For the piglet genotyping, the WUR SNP (WUR10000125) genotype, associated with PRRS tolerance (Boddicker et al., 2012; Boddicker et al., 2014; Hess et al., 2016), for each animal was included in our phenotypic dataset based on animals having either 0 or 1-2 copies of the favorable and assumed dominant allele (Boddicker et al., 2012). The effect of WUR SNP on the LS mean of piglet T3 for the 0 genotype was 36.4 ± 1.4 and for the 1 genotype was 38.5 ± 1.5, with a Prob > F = 0.0233 (Supplementary Data File S1). Only SNPs that were present on both versions of the genotyping platforms were retained for downstream analyses. SNPs were removed if they were unmapped or mapped to a sex chromosome in the swine genome build 11.1 (GenBank assembly accession: GCA_000003025.6) (latest), leaving in 58,563 SNPs. Quality control filtering was completed in PLINK software (Purcell et al., 2007); an animal was removed if it had a genotyping call rate less than 90%; SNPs that had a minor allele frequency (MAF) less than 1% were also removed. A total of 1792 animals and 54,357 SNPs were retained after quality control and used for downstream analyses.

For fetal genotyping, only SNPs that were present on both the Illumina Porcine SNP60 Beadchip version 2 and the Affymetrix Axiom Porcine Genotyping Array 650 K Beadchip were retained for downstream analyses. SNPs were matched between platforms using both RS identifiers and chromosomal map location, and MAF were verified between platforms. The WUR SNP was not included as a fixed effect in the fetal models as the impact of WUR on the fetal response to PRRS using the PGM model has been previously shown by our group to not be associated with response to infection (Ladinig et al., 2015). This resulted in 46,526 SNPs available for further processing. SNP processing was as noted for piglets and resulted in 1,267 fetuses (9 fetuses removed due to low genotyping call rate) and 38,843 SNPs used for downstream analyses.

#### 2.2.5 Genetic parameter estimation

The piglet and fetal genotype data were used separately to compute a genomic relationship matrix using the Van Raden Method (VanRaden, 2008). Genetic variance components and heritabilities were estimated using ASReml version 4 (Gilmour et al., 2002), separately for the piglet and fetal data sets. The models were developed using an iterative approach, fitting statistically and biologically relevant factors, as summarized in Supplementary Table S1. All fixed effects, covariates, and random effects included in the models were significant at *p* < 0.05. The Iowa State University High Performance Computing Nova cluster was used for these analyses. The following univariate mixed linear animal model was used for piglet_T3;

$$Y_{ijklmno}=\mu +T_{i}+S_{j}+W_{k}+V_{l}+TP_{m}+D_{n}+A_{o}+e_{ijklmno}$$

where Y is the phenotype of piglet T3 (ng/dL) at 11 DPI. Fixed effects included PHGC trial (T = 2, 4, 5, 7–15), sex (S = female, male, or barrow), and WUR SNP genotype (W = no copies or 1-2 copies of the favorable allele). Serum VL at 11 DPI (V) was included as a covariate to account for differences in infection levels (i.e., VL affects thyroid hormone levels). Random effects included pen nested within trial (TP), dam (i.e., litter) (D), and animal genetics (A). The residuals (e) were also included in the model.

The following univariate mixed linear animal model was used for both fetal_T3 and fetal_T4;

$$Y_{ijklmn}=\mu +T_{i}+S_{j}+F_{k}+V_{l}+D_{m}+A_{n}+e_{ijklmn}$$

where Y is the phenotype of fetal T3 (ng/dL) or T4 (µg/dL) at 12 or 21 DPMI. Fixed effects included PGM trial (T = PGM1 at 21 DPMI or PGM2 at 12 DPMI), sex (S = female or male), and fetal preservation status (F = viable, meconium staining on the head only, or meconium staining on the entire body). Thymus VL at 12 or 21 DPMI (V) was included as a covariate to account for differences in infection levels. Dam (D) and animal genetics (A) were fitted as random effects, along with residuals (e).

For all traits, the phenotypic variance was calculated as the sum of variance from dam, animal, and residuals. Heritability was calculated as the ratio of animal variance to phenotypic variance. Bivariate animal models were also run in ASReml to estimate phenotypic and genetic correlations between T3 and/or T4 traits and other important traits (i.e., WG and VL), using the same fixed and random effects as in the univariate models, and this applied to WG and VL traits as well as previously described (Hess et al., 2016).

#### 2.2.6 Genome-wide association analyses

GWAS for T3 and T4 levels during PRRSV infection were performed for each trait separately using the Julia for Whole-genome Analysis Software (JWAS) (Cheng et al., 2018). The ASREML genotypic and residual variance estimates were used as starting values in the JWAS analysis. In addition, the genomic relationship matrix results were included as input into the JWAS analysis. The Bayes-B model fits all SNPs simultaneously and, therefore, accounts for relationships and population structure and does not require additional inclusion of PCA or genomic relationships. The USDA-ARS SCINet High Performance Computing Ceres Cluster was used to run these analyses. The Bayes B approach (Habier et al., 2011) fits all SNPs simultaneously as random effects. We employed *π* = 0.999, and a Monte Carlo Markov Chain (MCMC) of length 50,000, with 5,000 for burn-in. The following mixed model was used for both the piglet and fetal datasets, with the same fixed and random effects as the univariate mixed linear animal model was used for piglet_T3, fetal_T3, and fetal_T4, respectively;

$$Y=Xb+Wu+\overset{k}{\underset{j}{\sum }}z_{j}\alpha_{j}\delta_{j}+e$$

Where ***Y*** is a vector of phenotypes, ***X*** is an incidence matrix for fixed effects in the model, ***b*** is a vector of fixed effects, ***W*** is an incidence matrix to account for other random effects in the model, ***u*** is a vector of random effects, ***z***_***j***_ is a vector of genotypes for SNP j based on the number of B alleles (0, 1, or 2), *α*_*j*_ is the allele substitution effect for SNP *j*, *δ*_*j*_ is a parameter that indicates whether SNP *j* was included in that sample of the MCMC, and ***e*** is the vector or residuals. The analyzed SNPs were split into 2,265 non-overlapping 1-Mb windows across the genome. Based on the infinitesimal model, each window is expected to explain 0.04% (100%/2265) of the genetic variation (GV). We considered windows that explained more than 1% of the GV as significant.

#### 2.2.7 Characterization of QTL regions

##### 2.2.7.1 Quantification of individual SNP effects and linkage disequilibrium

For each trait, the SNPs with the highest post-probability of inclusion (PPI) within the top 3 significant 1-Mb windows based on percentage of GV explained were investigated for impact on phenotypic traits. We fitted these SNP genotypes separately as fixed effects using univariate mixed linear animal model that was used for piglet_T3, fetal_T3, and fetal_T4, respectively in ASReml. The LS means and corresponding SE were estimated and pairwise T-tests between SNP genotypes were conducted with Tukey’s multiple testing correction; a *p* < 0.05 after multiple testing correction was considered significant. The linkage disequilibrium (LD) between SNPs that were within 1 Mb from the SNP with the highest PPI in the top 3 significant 1-Mb windows were estimated based on *r* ^2^, using the Plink software (Purcell et al., 2007).

##### 2.2.7.2 Candidate genes

For each trait, the SNP with the highest PPI was identified for each significant 1-Mb windows (i.e., ≥1% of the GV). Annotated genes that were located within 400 Kb (200 Kb upstream and 200 Kb downstream) of these SNPs were identified using ENSEMBL biomart (Howe et al., 2020) release 107 with the Pig - Duroc (Sscrofa11.1) option accessed on 30 August 2022. We chose this distance based on previously reported average LD blocks in commercial pig populations that were estimated to be 400 Kb in length (Veroneze et al., 2013).

## 3 Results

### 3.1 Genetic parameter estimates

Our investigations focused on serum thyroid hormone levels at 11 DPI in piglets, because T3 levels were most extremely suppressed at this timepoint post PRRSV challenge (Pasternak et al., 2021). Fetal T3 and T4 levels were measured on sera collected on the day of termination, at 12 or 21 DPMI. Descriptive statistics and heritability estimates of piglet and fetal traits are found in Table 1. Details on model factors included in the analyses are found in Supplementary Table S1. Heritability estimates for piglet_T3, fetal_T3, and fetal_T4 were lowly to moderately heritable. Heritabilities for economically (i.e., VL and WG-related measurements) and biologically (i.e., brain:liver ratio) relevant traits were estimated as well. These traits were further investigated for their phenotypic and genetic correlations with thyroid hormone levels. Piglet serum VL and fetal thymus VL were estimated to be moderately and lowly heritable, respectively. Piglet WG21 and WG42 were estimated to be moderately heritable, while fetal BW and brain:liver ratio on the day of euthanasia (i.e., 12 or 21 DPMI) were estimated to be moderately and lowly heritable, respectively.

**TABLE 1:** Descriptive statistics, heritability, and variance (dam, animal, residual, and total phenotypic) estimates of piglet and fetal traits.

Trait	N	Mean (SE)	Min	Max	h^2^ (SE)	Dam var. est	Animal var. est	Residual var. est	Total phenotypic var. est
Piglets									
T3^a^	1792	38.64 (0.41)	2.12	167.75	0.16 (0.05)	18.12 (5.90)	37.50 (10.60)	149.12 (7.98)	232.91 (9.49)
VL^b^	1792	5.70 (0.02)	3.02	7.44	0.34 (0.06)	0.40E-01 (0.73E-02)	0.65E-01 (0.14E-01)	0.13 (0.85E-02)	0.19 (0.11E-01)
WG21^c^	1,577	6.58 (0.07)	−2.4	21.1	0.43 (0.06)	0.47 (0.14)	2.29 (0.40)	3.04 (0.21)	5.33 (0.31)
WG42^d^	1,293	19.04 (0.15)	1.09	53.9	0.35 (0.06)	1.80 (0.69)	7.13 (1.63)	13.27 (0.97)	20.40 (1.30)
Fetuses									
T3^e^	1,187	34.99 (0.43)	4.93	114.60	0.10 (0.06)	39.37 (8.65)	17.52 (10.18)	123.48 (7.02)	180.37 (9.45)
T4^f^	1,187	2.34 (0.03)	0.22	8.06	0.10 (0.06)	0.17 (0.04)	0.09 (0.06)	0.73 (0.04)	1.00 (0.04)
VL^g^	1,187	2.34 (0.09)	0	8.80	0.09 (0.06)	1.42 (0.28)	0.47 (0.36)	4.98 (0.28)	5.46 (0.30)
BW^h^	1,187	902.0 (7.7)	63.8	1861.0	0.57 (0.05)	39.93 (8.78)	35822.30 (4455.51)	27112.1 (2129.78)	62934.00 (3527.80)
Brain:liver^i^	1,184	1.20 (0.01)	0.39	3.83	0.05 (0.08)	0.04 (0.71E-02)	0.20E-02 (0.80 E−02)	0.94E-01 (0.54E-02)	0.96E-01 (0.61E-02)

^a^ Triiodothyronine (T3) levels were measured in serum at 11 DPI, ng/dL

^b^ Viral load (VL) was measured in serum at 11 DPI, log10viral load.

^c^ Body weight gain (WG21, kg) was calculated as the difference between body weight at 0 and 21 DPI. Note animals from trial 9 were excluded from the dataset prior to these calculations.

^d^ Body weight gain (WG42, kg) was calculated as the difference between body weight at 0 and 42 DPI. Note animals from trial 9 were excluded from the dataset prior to these calculations, and animals from trials 7 and 8 were not measured at 42 DPI due to accessibility of the facility.

^e^ T3 levels (ng/dL) were measured in serum at 12 or 21 DPMI.

^f^ Thyroxine (T4) levels (µg/dL) were measured in serum at 12 or 21 DPMI.

^g^ VL (log10viral load) was measured in the fetal thymus at 12 or 21 DPMI.

^h^ Fetal body weight (BW) (grams) was measured at 12 or 21 DPMI.

^i^ Fetal brain to liver weight ratios were calculated based on weights measured at 12 or 21 DPMI.

Estimates of phenotypic and genetic correlations between T3, VL, WG21 and WG42 for piglets are presented in Table 2. Piglet_T3 had a negative moderate genetic correlation (r_g_) with serum VL (r_g_ = −0.34). Moreover, there was a positive high genetic correlation between piglet_T3 and both WG21 and WG42 with r_g_ = 0.81 and r_g_ = 0.67, respectively. Our results were consistent (within the margins of the SE) as those previously calculated as high genetic correlations between serum VL and WG21 or WG42 (Boddicker et al., 2012) with r_g_ = −0.35 and r_g_ = −0.27, respectively.

**TABLE 2:** Estimates of phenotypic (below diagonal) and genetic (above diagonal) correlations (SE) between thyroid hormone level, VL, WG21, and WG42 in piglets.

Trait	T3	VL	WG21	WG42
T3^a^		−0.34 (0.14)	0.81 (0.09)	0.67 (0.14)
VL^b^	−0.23 (0.04)		−0.35 (0.12)	−0.27 (0.14)
WG21^c^	0.36 (0.03)	−0.20 (0.04)		0.97 (0.03)
WG42^d^	0.26 (0.03)	−0.27 (0.04)	0.86 (0.01)	

^a^ Triiodothyronine (T3, ng/dL) levels were measured in serum at 11 DPI.

^b^ Viral load (VL, log10viral load) was measured in serum at 11 DPI.

^c^ Body weight gain (WG21, kg) was calculated as the difference between body weight at 0 and 21 DPI. Note animals from trial 9 were excluded from the dataset prior to these calculations.

^d^ Body weight gain (WG42, kg) was calculated as the difference between body weight at 0 and 42 DPI. Note animals from trial 9 were excluded from the dataset prior to these calculations, and animals from trials 7 and 8 were not measured at 42 DPI due to accessibility of the facility.

Estimates of phenotypic and genetic correlations among fetal T3, T4, VL, BW, and brain:liver ratios are presented in Table 3. We found moderate genetic correlation estimates between fetal_T3 and fetal_T4 levels. Negative moderate to high genetic correlations were estimated of fetal_T3 and fetal_T4 with thymus VL. The positive genetic correlation between fetal_T4 with fetal BW was estimated to be much higher compared to that between fetal_T3 with fetal BW. Negative high genetic correlations were estimated for both fetal_T3 and fetal_T4 with brain:liver ratios; however, very large SE were associated with these estimates. The estimate of the genetic correlation of fetal thymus VL with fetal BW was moderately positive (i.e., increased VL associated with increased BW) but had a large SE as well. The estimate of the genetic correlation of fetal thymus VL with brain:liver ratio was high negative but also had a large SE.

**TABLE 3:** Phenotypic (below diagonal) and genetic (above diagonal) correlation coefficients (SE) between fetal thyroid hormone levels, VL, BW, and brain:liver ratio.

Traits	T3	T4	VL	BW	Brain:liver
T3^a^		0.48 (0.21)	−0.84 (0.38)	0.10 (0.20)	−0.99 (1.90)
T4^b^	0.39 (0.03)		−0.77 (0.26)	0.67 (0.17)	−0.79 (1.74)
VL^c^	−0.38 (0.04)	−0.35 (0.04)		0.44 (0.41)	−0.97 (0.78)
BW^d^	−0.01 (0.04)	0.26 (0.04)	0.12 (0.05)		−0.83 (0.92)
Brain:liver^e^	−0.16 (0.04)	−0.19 (0.04)	−0.25 (0.05)	−0.71 (0.12)	

^a^ Triiodothyronine (T3, ng/dL) levels were measured in serum at 12 or 21 DPMI.

^b^ Thyroxine (T4, µg/dL) levels were measured in serum at 12 or 21 DPMI.

^c^ Viral load (VL, log10viral load) was measured in the fetal thymus at 12 or 21 DPMI.

^d^ Fetal body weight (BW, grams) was measured at 12 or 21 DPMI.

^e^ Fetal brain to liver ratios were calculated based on weights measured at 12 or 21 DPMI.

### 3.2 Genome-wide association analyses

Detailed GWAS results for piglet_T3, fetal_T3, and fetal_T4 are found in Table 4, with information for each 1-Mb window that explained more than 1% of the total GV and the SNP within each window that had the highest PPI. Significant consecutive windows were designated as a single QTL. For example, we identified two significant windows on SSC5 for Piglet_T3 in the 69 and 70 Mb position that explained 11.37% and 3.32% of the GV, respectively. These two significant windows were considered a single QTL that explained 14.69% of the GV, rather than two QTL.

**TABLE 4:** Summary of GWAS results for piglet_T3, fetal_T3, and fetal_T4 with SNP information for each 1-Mb window with more than 1% of the GV.

Window					SNP with highest PPI within the window				
Trait^a^	Chr^b^	Pos (Mb)^c^	% GV	Num of SNPs	SNP name^d^	SNP pos (bp)^e^	PPI^f^	MAF^g^	Num of genes^h^
Piglet_T3	6	35	3.13	17	ALGA0035153	35,040,992	0.38	0.47	1
Piglet_T3	3	129	3.33	27	MARC0089811	129,729,248	0.33	0.27	1
Piglet_T3	5	69	11.37	34	INRA0019871	69,976,623	0.33	0.46	5
Piglet_T3	5	70	3.32	20	H3GA0016790	70,032,357	0.19	0.46	6
Piglet_T3	4	109	1.08	27	H3GA0014196	109,681,421	0.17	0.1	7
Piglet_T3	15	49	1.11	12	ALGA0085350	49,434,699	0.15	0.2	1
Piglet_T3	17	17	1.96	34	ALGA0093511	17,413,225	0.15	0.46	1
Piglet_T3	7	25	1.27	37	ASGA0032123	25,400,951	0.14	0.21	9
Piglet_T3	14	89	1.45	32	MARC0089051	89,227,581	0.1	0.18	3
Piglet_T3	14	90	1.21	32	ALGA0079862	90,282,613	0.1	0.2	5
Piglet_T3	15	117	0.98	28	ALGA0086944	117,536,030	0.09	0.01	3
Fetal_T3	1	253	1.25	33	ALGA0009315	252,973,691	0.06	0.16	4
Fetal_T3	4	112	4.67	32	ALGA0028303	112,497,789	0.04	0.12	0
Fetal_T3	4	113	1.26	25	ALGA0119977	1,13,519,433	0.03	0.18	0
Fetal_T3	1	254	1.68	27	ALGA0009329	253,112,371	0.02	0.13	3
Fetal_T3	4	83	1.21	16	ALGA0026269	83,435,243	0.02	0.49	5
Fetal_T4	1	155	1.5	9	ALGA0116832	155,713,656	0.07	0.42	2
Fetal_T4	10	48	1.06	12	MARC0079247	48,363,545	0.07	0.11	7
Fetal_T4	13	29	3.51	26	ASGA0057067	29,857,627	0.07	0.34	6
Fetal_T4	15	117	1.92	17	DIAS0000510	117,674,402	0.06	0.38	2
Fetal_T4	6	2	1.5	14	ASGA0093316	2,853,231	0.05	0.2	1
Fetal_T4	15	120	1.44	21	ASGA0070586	120,106,066	0.04	0.38	3
Fetal_T4	13	30	1.08	14	SIRI0000359	30,413,447	0.03	0.42	5
Fetal_T4	15	31	1.54	25	MARC0052461	31,397,274	0.03	0.43	13

^a^ Triiodothyronine (T3) levels were measured in piglet serum at 11 DPI (piglet_T3), in fetal serum at 12 or 21 DPMI (fetal_T3); thyroxine (T4) levels were measured in fetal serum at 12 or 21 DPMI (fetal_T4).

^b^ Chromosome (Chr) where a significant window was identified based on *Sus scrofa* 11.1 (Sscrofa11.1) build.

^c^ Position (Pos) in megabases (Mb) on given chromosome where significant window was identified based on Sscrofa11.1 build.

^d^ SNP name based on Illumina Porcine SNP60 Beadchip version 2 nomenclature.

^e^ SNP location based on SSC Sscrofa11.1 build.

^f^ Posterior Probability of Inclusion (PPI): frequency with which the SNP was included in the MCMC iterations (post-burn-in).

^g^ Minor allele frequency (MAF) of the SNP within the genotyped populations (N = 1792 animals for piglet_T3, N = 1,187 animals for both fetal traits).

^h^ Number of annotated candidate genes within 400 Kb (200 Kb upstream and 200 Kb downstream) of SNP based on Ensembl Biomart release 107 with the Pig—Duroc (Sscrofa11.1) option accessed on 30 August 2022.

**FIGURE 1:**
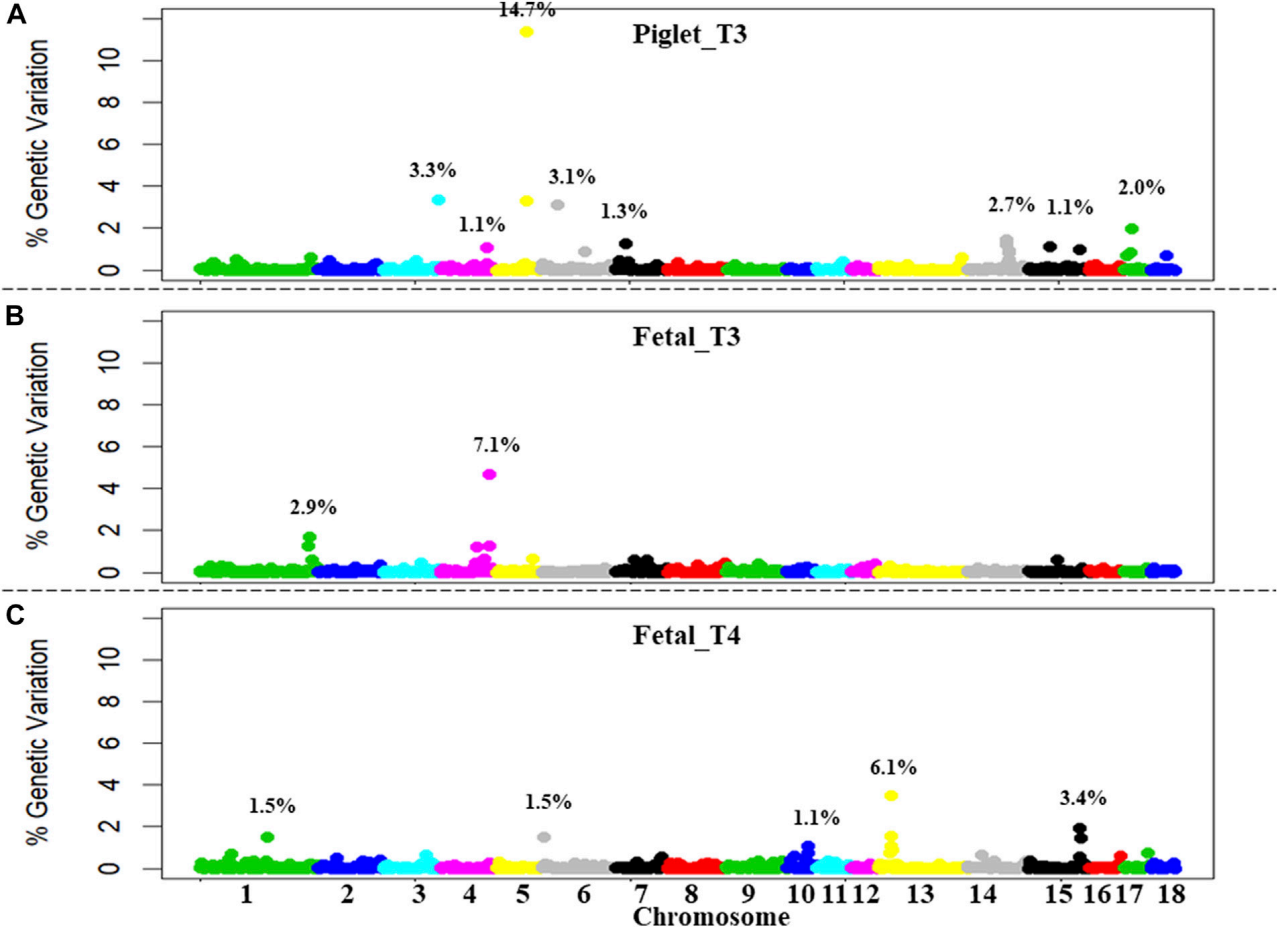
Genome-wide association study (GWAS) plot of the percentage of GV for thyroid hormone levels measured following PRRSV2 challenge in piglets and fetuses. **(A)** Triiodothyronine (T3) levels were measured in piglet serum at 11 DPI (piglet_T3). **(B)** T3 levels were measured in fetal serum at 12 or 21 DPMI (fetal_T3). **(C)** Thyroxine (T4) levels were measured in fetal serum at 12 or 21 DPMI (fetal_T4). Results show the percentage of GV from 0% to 12% explained by each non-overlapping 1-Mb window. Windows are ordered, labeled, and colored by chromosome (SSC1-18). Significant QTL (1 or more consecutive windows explaining ≥1% of the genetic variation each) are highlighted with the summation of total GV explained.

Nine QTL were identified for piglet_T3 (Figure 1A) in a total of eleven significant 1-Mb windows. These windows collectively explained 30% of the total GV. The two consecutive windows identified on SSC5 explained 15% of the GV alone. Three QTL were identified for fetal_T3 (Figure 1B) in a total of five significant 1-Mb windows. These windows collectively explained 10% of the GV. The two consecutive windows identified on SSC4 explained 6% of the GV alone. Six QTL were identified for fetal_T4 (Figure 1C) in a total of eight significant 1-Mb windows. These windows collectively explained 14% of the GV. The three consecutive windows identified on SSC13 explained 6% of the GV alone. QTL co-localizations were identified within 3 Mb on SSC4 (piglet_T3 and fetal_T3), within 33-Mb on SSC6 (piglet_T3 and fetal_T4), and within the same 1-Mb window on SSC15 (piglet_T3 and fetal_T4).

### 3.3 Characterization of QTL regions

#### 3.3.1 Quantification of individual SNP effects and linkage disequilibrium

**FIGURE 2:**
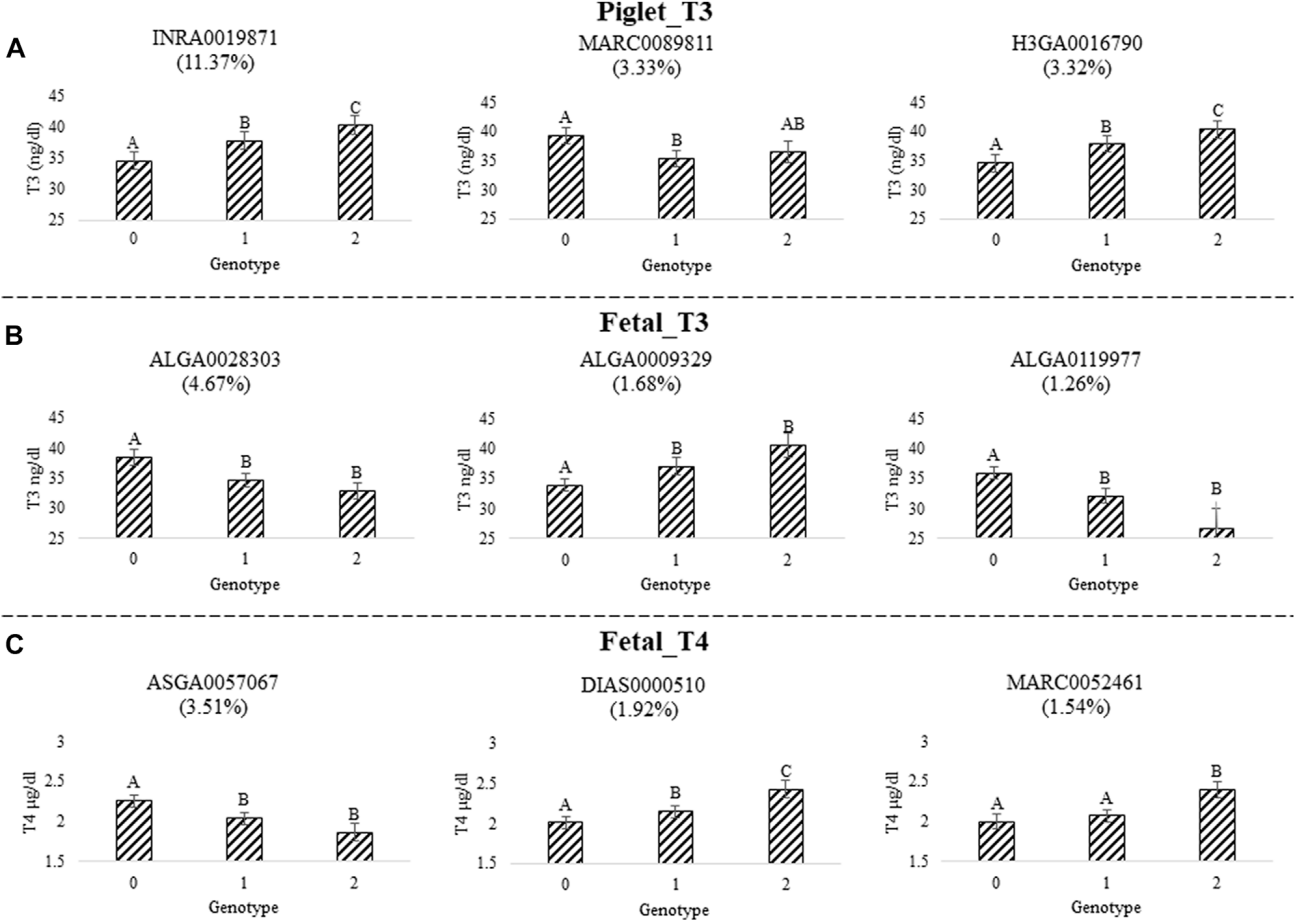
Top three SNP effects for serum thyroid hormone levels measured during challenge with PRRSV2. **(A)** Triiodothyronine (T3) levels were measured in piglet serum at 11 DPI (piglet_T3). SNP INRA0019871, MARC0089811, and H3GA0016790 were in windows that explained 11.37%, 3.33%, and 3.32% of the GV, respectively. **(B)** T3 levels were measured in fetal serum at 12 or 21 DPMI (fetal_T3). SNP ALGA0028303, ALGA0009329, and ALGA0119977 were in windows that explained 4.67%, 1.68%, and 1.26% of the GV, respectively. **(C)** Thyroxine (T4) levels were measured in fetal serum at 12 or 21 DPMI (fetal_T4). SNP ASGA0057067, DIAS0000510, and MARC0052461 were in windows that explained 3.51%, 1.92%, and 1.54% of the GV, respectively. The results show the top 3 windows for each trait represented by the top SNP. Plots depict the LS mean ± SE of thyroid hormone levels by SNP genotype. Different letters indicate *p* < 0.05 after multiple testing correction. The top SNP was determined based on the SNP with the highest PPI within each of the top three 1-Mb windows that explained the largest amount of GV.

We quantified the impact of the top three SNPs for each trait based on PPI in the 1-Mb window that explained the largest amount of GV for each trait. We fit univariate mixed models and included the individual SNP genotype in the model as a fixed effect. We found that all 9 SNPs (i.e., 3 for each trait) were highly significantly associated with thyroid hormone levels (*p* < 0.0001). The least-square (LS) means for each SNP genotype were calculated and the statistical difference between individual genotypes were determined (Figure 2). Details for piglet_T3, fetal_T3, and fetal_T4 are found in Figures 2A–C, respectively. All 9 SNPs had at least one significant difference (*p* < 0.05) for thyroid hormone levels between animals with different genotypes at the individual SNP. Three of these SNPs had patterns of additive inheritance (INRA0019871, H3GA0016790, and DIAS0000510), whereas five showed the homozygous recessive was needed for a favorable phenotype (MARC0089811, ALGA0028303, ALGA0119977, ASGA0057067, and MARC0052461), and one of these SNPs had a pattern of dominance inheritance (ALGA00009329).

The LD estimates (*r* ^2^) were calculated for the top SNP (±1 Mb) within all significant windows (Supplementary Table S2). Overall, we found an average *r* ^2^ value of 0.21 and a median of 0.11. High LD with *r* ^2^ of ≥0.80 on average extended over 0.19 Mb in distance, consistent with our candidate gene search within ±0.2 Mb from the top SNPs. For the consecutive significant windows for piglet_T3 on SSC5 and SSC14, we found *r* ^2^ values of 0.33 and 0.26, respectively. For consecutive significant windows identified for fetal_T3 on SSC1 and SSC4, we found *r* ^2^ values of 0.16 and 0.26, respectively. For consecutive significant windows identified for fetal_T4 on SSC13, we found *r* ^2^ of 0.38.

#### 3.3.2 Candidate genes

For each trait, positional candidate genes were identified within 400 Kb (200 Kb upstream and 200 Kb downstream) of the top SNP in each significant window. The top SNP was determined based on the SNP with the highest PPI within each of the 1-Mb windows that explained >1% of the GV. Results for all significant SNP revealed a total of 93 annotated genes, enumerated in Table 4; more details for all 93 genes are found in Supplementary Table S3. For the top 3 SNP for each trait, we identified 12, 3, and 21 total positional candidate genes for piglet_T3, fetal_T3, and fetal_T4, respectively and are detailed in Table 5. Genes varied in function but generally fell into the categories of immune-related, transporter-related, and transcriptional regulators.

**TABLE 5:** Positional candidate genes for the top SNP based on percentage of GV explained for each trait.

Trait^a^	SNP (% GV)^b^	Chr^c^	Gene name^d^	Description
Piglet_T3	INRA0019871 (11.37%)	5	*BCL2L13*	BCL2 like 13
			*BID*	BH3 interacting domain death agonist
			*MICAL3*	Microtubule associated monooxygenase, calponin and LIM domain containing 3
			*PEX26*	Peroxisomal biogenesis factor 26
			*TUBA8*	Tubulin alpha 8
Piglet_T3	MARC0089811 (3.33%)	3	*SOX11*	SRY-box transcription factor 11
Piglet_T3	H3GA0016790 (3.32%)	5	*BCL2L13*	BCL2 like 13
			*BID*	BH3 interacting domain death agonist
			*MICAL3*	Microtubule associated monooxygenase, calponin and LIM domain containing 3
			*PEX26*	Peroxisomal biogenesis factor 26
			*TUBA8*	Tubulin alpha 8
			*USP18*	Ubiquitin specific peptidase 18
Fetal_T3	ALGA0028303 (4.67%)	4	None	N/A
Fetal_T3	ALGA0009329 (1.68%)	1	*HSDL2*	Hydroxysteroid dehydrogenase like 2
			*PTBP3*	Polypyrimidine tract binding protein 3
			*SUSD1*	Sushi domain containing 1
Fetal_T3	ALGA0119977 (1.26%)	4	None	N/A
Fetal_T4	ASGA0057067 (3.51%)	13	*CCDC12*	Coiled-coil domain containing 12
			*KIF9*	Kinesin family member 9
			*MYL3*	Myosin light chain 3
			*NBEAL2*	Neurobeachin like 2
			*PTH1R*	Parathyroid hormone 1 receptor
			*SETD2*	SET domain containing 2, histone lysine methyltransferase
Fetal_T4	DIAS0000510 (1.92%)	15	*ATIC*	5-aminoimidazole-4-carboxamide ribonucleotide Formyltransferase/IMP cyclohydrolase
			*FN1*	Fibronectin 1
Fetal_T4	MARC0052461 (1.54%)	15	*ARIH2*	Ariadne RBR E3 ubiquitin protein ligase 2
			*CELSR3*	Cadherin EGF LAG seven-pass G-type receptor 3
			*IP6K2*	Inositol hexakisphosphate kinase 2
			*NCKIPSD*	NCK interacting protein with SH3 domain
			*PFKFB4*	6-phosphofructo-2-kinase/fructose-2,6-biphosphatase 4
			*PRKAR2A*	Protein kinase cAMP-dependent type II regulatory subunit alpha
			*SHISA5*	Shisa family member 5
			*SLC25A20*	Solute carrier family 25 member 20
			*SLC26A6*	Solute carrier family 26 member 6
			*TMEM89*	Transmembrane protein 89
			*U6*	U6 spliceosomal RNA
			*UCN2*	Urocortin 2
			*UQCRC1*	Ubiquinol-cytochrome c reductase core protein 1

^a^ Triiodothyronine (T3) levels were measured in piglet serum at 11 DPI (piglet_T3), in fetal serum at 12 or 21 DPMI (fetal_T3); thyroxine (T4) levels were measured in fetal serum at 12 or 21 DPMI (fetal_T4).

^b^ SNP name based on Illumina Porcine SNP60 Beadchip version 2 nomenclature and percent genetic variation (% GV) that was explained by the 1-Mb window for which the SNP was identified as having the highest PPI in the MCMC iterations.

^c^ Chromosome (Chr) where a significant window was identified based on *Sus scrofa* 11.1 (Sscrofa11.1) build.

^d^ Gene name based on Ensembl Biomart release 107 with the Pig—Duroc genes (Sscrofa11.1) option accessed on 30 August 2022.

## 4 Discussion

Our study estimated genetic parameters, identified QTL, and characterized QTL regions for thyroid hormone (i.e., T3 and/or T4) levels in piglets and fetuses challenged with PRRSV2. Our main findings were: 1) thyroid hormone levels following PRRSV infection were lowly to moderately heritable in piglets and fetuses; 2) thyroid hormone levels had negative moderate to high genetic correlations with VL and positive moderate to high genetic correlations with weight-related traits, both economically important disease resilience traits; 3) QTL were identified and the significant 1-Mb windows collectively explained a large amount of the GV. These windows were identified based on individual SNP that had the highest PPI within significant 1-Mb windows for thyroid hormone levels; and 4) biologically relevant candidate genes were identified near SNP markers that had the highest PPI within significant 1-Mb windows. These results advance our understanding of both piglet and fetal response to PRRSV infection, bringing us one step further to characterizing the genomic control of phenotypes associated with host resilience and susceptibility.

### 4.1 Thyroid hormones levels during disease challenge are heritable

Estimates of heritability of piglet_T3, fetal_T3, and fetal_T4 during PRRSV2 challenge were 16%, 10%, and 10%, respectively. We chose to use the animal model for both piglet and fetal traits with dam included as an independent random effect to account for common environmental effects. The direct-maternal genetic model was not employed for fetal traits due to the absence of pedigree records or genotypes of dams. We hypothesize that heritability of this response phenotype is greater in the piglet samples from the PHGC trials due to the fixed inoculation time compared with the more complex dynamics associated with maternal challenge and transplacental infection in the reproductive PGM trials. Furthermore, while the porcine placenta is known to act as an enzymatic barrier for thyroid hormone (Krysin et al., 1997), maternal contribution under disease conditions cannot be entirely excluded. However, recent investigation of thyroid hormone metabolism within the maternal fetal interface of PRRSV challenged fetuses identified decompensatory expression of deiodinases which would further limit vertical transmission (Ison et al., 2022).

Serum thyroid hormone levels in humans during periods of homeostasis have been estimated to be moderately to highly heritable, at 23%–64% and 28%–65% for T3 and T4, respectively (Meikle et al., 1988; Hansen et al., 2004; Panicker et al., 2008). There have been far fewer studies on the genetic influence of thyroid hormone levels in food animals. A recent study in Holstein cattle estimated the heritability of T3 and T4 at 11% and 19%, respectively (Gan et al., 2020). The heritability estimates conducted in non-challenged cattle are similar to those identified in the current study, within the margin of SE. The lower heritability estimates for serum thyroid hormone levels of swine, found here compared to humans, may be due to our data being collected during disease challenge with PRRSV. Moreover, in humans it has been reported that intra-individual variation in thyroid hormone levels is much lower than inter-individual variation, which may suggest that disease state levels of thyroid hormones may be largely dependent on an individual’s normal set-point for thyroid hormone levels (Andersen et al., 2002). In the current study, we investigated thyroid hormone levels at 11 DPI in piglets and at 12 or 21 DPMI in fetuses. We chose 11 DPI for piglet samples because thyroid hormone levels were most extremely suppressed at this timepoint post PRRSV challenge (Pasternak et al., 2021). Opportunity exists for future studies in growing pigs to investigate thyroid hormone levels expressed as the differential between pre-challenge and post-challenge levels within an individual animal. This approach would assess the impact of the individual animal’s thyroid hormone level set-point and could give further insight into host resilience. However, this approach may not be feasible in fetuses due to the cost and logistical complexity of collecting serial samples in fetuses *in utero*.

In the current study, we also estimated heritabilities for important resilience and production measurements; viral levels and weight-related traits. In the piglet_T3 dataset, we estimated moderate heritabilities for serum VL at 11 DPI, WG21, and WG42 to be 34%, 43%, and 35%, respectively. These estimates replicate those previously reported and are within the margin of SE on the full PHGC dataset (Boddicker et al., 2012). In the fetal_T3 and T4 datasets, we estimated low to high heritabilities of fetal thymus VL, fetal BW, and brain:liver ratio at 9%, 57%, and 5%, respectively. To our knowledge, our study is the first to report heritability estimates for fetal VL during PRRSV infection in swine. Swine birth weight in a non-challenge model has been previously reported to have much lower heritability than in our study, ranging from 8% to 12% (Damgaard et al., 2003; Wittenburg et al., 2008). Our heritability estimates for fetal BW could be influenced by the population of dams within the study, although we accounted for the dam effect by fitting it as a random effect in the model. In contrast, the estimate of heritability for brain:liver ratio was quite low, with a SE that was higher in magnitude than the estimate (0.05 ± 0.08), which support previously reported low estimates of heritability in non-challenged fetuses (0.01 ± 0.01) (Matheson et al., 2018). Previous PRRS research has demonstrated that fetuses with lower BW (i.e., classified as IUGR fetuses) have lower VL and are less frequently compromised or die *in utero*, compared to non-IUGR fetuses (Ladinig et al., 2014a; Malgarin et al., 2019). Our data confirm that non-genetic factors have substantially more impact on brain:liver ratio (i.e., a IUGR measurement) than genetic factors, and therefore may not be a good candidate trait for selection.

### 4.2 Thyroid hormone levels have moderate to high genetic correlations with other disease resilience traits

In the current study we estimated phenotypic and genetic correlations of thyroid hormone levels with VL and weight-related traits. Thyroid hormone levels and associated HPT axis products have been shown to be altered due to feed intake (Buonomo and Baile, 1991), diet (Spiegel et al., 1993; Carroll et al., 1998), and in response to pathogen or antigen challenge (Castro et al., 2013; Pasternak et al., 2020b; Pasternak et al., 2021). Therefore, we anticipated identifying phenotypic correlations between these traits, which was confirmed in our study (Tables 2 and 3). To our knowledge, we are the first to report genetic correlations between thyroid hormone levels and VL and weight-related traits in swine. For piglet_T3 we found moderate to high genetic correlations of −0.34, 0.81, and 0.67 with VL, WG21, and WG42, respectively. These correlations indicate that selection for higher T3 levels would indirectly select for lower VL and higher WG under challenge, which are key traits of resilience to PRRSV infection in the nursery pig model (Dekkers et al., 2017).

We found a moderate positive genetic correlation between fetal_T3 and fetal_T4 levels of 0.48, indicating the genetic selection could target them independently due to apparently distinct genetic control of their levels during PRRSV infection. We expected to estimate an even larger genetic correlation due to the biological sources of these hormones. The thyroid gland mainly produces and releases T4 which is converted by local deiodinase enzymes within peripheral tissues to T3, the more bioactive form. This relationship could lead to a negative phenotypic correlation if more T4 is converted to T3, which reduces the level of T4 and increases the level of T3. T3 then initiates cellular response by binding the nuclear receptors, THRa and THRb, to directly regulate gene expression. A recent study identified THRb as a candidate gene near a QTL for the reproductive trait total number born (Chang Wu et al., 2022), further linking the importance of these pathways in fetal outcome. Alternatively, T4 can be converted to other non-bioactive metabolites including reverse triiodothyronine (rT3) and various diiodothyronines (T2) by similar enzyme activity in the liver, kidney, or placenta (Krysin et al., 1997; van der Spek et al., 2017). Previous work from our group on PRRSV infected fetal thymus and placenta showed downregulation of gene expression for thyroid hormone receptors (*THRa* and *THRb*), along with the critical outer ring deiodinase (*DIO2*), required to convert T4 to the more bioactive T3 (Van Goor et al., 2020). Thus, there is complex regulation of both hormones.

We chose to investigate piglet T3 in serum, but not piglet T4, due to sample assay restraints at the time. Future research should investigate piglet T4 levels during challenge given the distinct genetic control compared to T3 that was identified for fetal T3 and T4 levels in the current study. We found both fetal_T3 and T4 to have strong negative genetic correlations with VL at −0.77 and −0.84, respectively. However, all three of these traits have low heritabilities (0.09–0.10) which suggests that large gains in selection would be difficult. Genetic correlations of the fetal brain:liver ratio with other traits were estimated as very high (−0.79 to −0.99) but were largely uninformative due to the large SE and low heritability of this trait. However, this may be data dependent. Here, we used samples from the largest reproductive PRRS challenge models with a total of 1,184 fetuses analyzed for brain:liver ratios. Potential exists to add more fetal phenotypes to reduce SE and potentially capture more GV, given the uniqueness of this trait. In contrast, we identified a moderate positive genetic correlation between fetal_T4 with BW (0.67), indicating animals with higher levels of T4 could be selected to increase BW of fetuses. We expected, and confirmed, a positive phenotypic correlation between fetal VL and BW and a negative correlation between fetal VL and brain:liver ratio. Previous research reports IUGR fetuses (i.e., smaller “brain-spared” fetuses with large brain:liver weight ratios) tend to have lower VL compared average growth fetuses (Ladinig et al., 2014a). Our study focused on thyroid hormone levels during disease challenge and previous works indicate the strong decrease of both T3 and T4 levels after PRRSV infection (Pasternak et al., 2020b; Pasternak et al., 2021). Our data support the concept that T3 and/or T4 levels during challenge may be good measures of resilience, as the animals that are able to maintain higher thyroid hormone levels during challenge are also able to reduce VL and maintain higher weight-related measurements, each of which are controlled by some of the same genetic regions.

### 4.3 Thyroid hormone levels are influenced by relatively few QTL with large effects

Here we report for the first time QTL identified for thyroid hormone levels in swine. Our study found a total of 18 QTL across 11 chromosomes for T3 and/or T4 levels during challenge with PRRSV, collectively explaining 10%–31% of the GV for a trait. The GWAS analyses were completed in JWAS software partly because of the option to fit additional random effects, that could be biologically important (e.g., dam), in the model compared to other software (e.g., GenSel). The Pig QTL Database containing 34,793 QTL was used to identify relevant (previously identified) nearby (±2 Mb) QTL with the top three significant regions identified in the current study (i.e., piglet_T3 on SSC3 and 5, fetal_T3 on SSC1 and 4, fetal_T4 on SSC13 and 15).

A QTL for piglet_T3 was found on SSC3, explained 3% of the GV, and co-localized with a previously identified QTL for PRRS VL in the host using an expanded dataset from the PHGC (Waide et al., 2017). This region may be an important candidate for genetic selection of resilience as measured by both T3 and VL. The largest QTL among all traits in the current study was identified for piglet_T3 located on SSC5 that had two consecutively significant 1-Mb windows, which explained 15% of the GV alone. The SNPs with highest PPI in these windows were located at 69976623bp and 70032357bp.There were a total of 117 reported QTL in this region; the QTL on SSC5 co-localized with a previously identified QTL for WG42 during PRRSV infection in the host using an expanded dataset from the PHGC (Waide et al., 2017). In addition, two previously reported QTL in this region are related to immune measurements, including white blood cell count (Cho et al., 2011) and haptoglobin concentration (Wimmers et al., 2009). There is a delicate balance between resource allocation to utilize energy to fight an infection *versus* to use for growth. The overlap between QTL for thyroid hormone levels and adaptive immunity traits identified here may indicate animals were able to successfully initiate adaptive immunity while maintaining sufficient levels of thyroid hormone during challenge.

A QTL for fetal_T3 that explained 3% of the GV was identified on SSC1 that had two consecutively significant 1-Mb windows. The SNPs with highest PPI in these windows were located at 252973691bp and 253112371bp. This QTL overlapped with 145 previously reported QTL, and co-localized with a QTL for mean corpuscular hemoglobin concentration identified in a study investigating immune-related traits in two pig lines (Dauben et al., 2021). Reproductive traits, compared to growth-related traits, are more difficult to select for because they have low heritability and are typically controlled by many genes each with small effects (Zak et al., 2017). The region on SSC1 has previously been reported to contain a QTL for gestational length (Wilkie et al., 1999). It is likely that a link may exist between reproduction traits and fetal response to infection due to the complex environment of the fetus. This region on SSC1 also contained three previously identified QTL for relative number of leukocytes (Wattrang et al., 2005; Reiner et al., 2008; Cho et al., 2011). Previous work has documented the association between leukocyte subsets in pregnant gilts infected with PRRSV and fetal response to infection (Ladinig et al., 2014b). In addition, a QTL for PRRS VL in the fetal thymus was previously identified on SSC1 (Yang et al., 2016), near the QTL identified for fetal_T3 in the current study, and explained 6% of the GV. However, we fitted fetal thymus VL as a covariate in our GWAS model to account for differences in infection level (i.e., VL affects thyroid hormone levels), and the two QTL (i.e., a QTL identified in the current study and the previously identified QTL for PRRS VL) were ∼50 Mb apart in distance. Moreover, we calculated the average LD block in our dataset as ∼200 Kb in length. Although it is possible, it is unlikely that the same SSC1 genes are impacting both traits.

We found overlapping QTL on SSC4 between 109 and 113 Mb for both fetal_T3 and piglet_T3 levels. A total of 264 QTL are reported in the Pig QTL database within this region (i.e., ±2 Mb), including several for average daily gain (Knott et al., 2002), birth weight (Walling et al., 2000), preweaning failure to thrive syndrome (Bertolini et al., 2018), and coping behavior (Ponsuksili et al., 2015), an indicator of stress tolerance. The overlapping QTL may help identify similarities in the genetic architecture between T3 levels in both the piglet and fetal challenge models. There are seven genes within 200 Kb of the top SNP within each significant window for the traits (Supplementary Data File S3). These genes were all identified nearest the QTL on SSC4 for piglet T3 (i.e, H3GA0014196 at 109681421bp). These include *KCNA2*, *KCNA3*, *KCNA10*, *LAMTOR5*, *PROK1*, *RBM15*, and *SLC16A4*. Potassium voltage-gated channel gene expression have previously been shown to change in expression in the heart in response to hypothyroidism in rats (Nishiyama et al., 1998). Previous research using the PGM has identified changes in gene expression in fetal hearts following PRRSV infection (Malgarin et al., 2021). The LAMTOR5 protein is a late endosomal/lysosomal adaptor, MAPK and MTOR activator, which are extremely important immune response pathways and are addressed below. Some members of the solute carrier transmembrane transporters have been shown to aid in the traffic of T3 and T4 from the serum into the cytoplasm (Krause and Hinz, 2020). Although *SLC16A4* has not been implicated in thyroid hormone interaction, future research could investigate this gene for the potential role. Future research should investigate the genes identified here to help uncover phenotypes that could aid in our understanding of both piglet and fetal T3 response to PRRSV infection.

One of the most important findings of the PHGC was identification of the WUR genetic marker on SSC4, which explained a large proportion of GV for both VL and WG in PRRSV infected piglets (Boddicker et al., 2012; Boddicker et al., 2014; Hess et al., 2016) but not for fetuses (Yang et al., 2016). The WUR SNP genotype was included as a fixed effect in the piglet_T3 analyses here; the WUR SNP was present in both the piglet and fetal datasets that were used for GWAS analyses. In the newest swine genome 11.1 build, WUR is located 14 Mb (SSC4:127,441,677) away from the SSC4 QTL identified here for piglet_T3 and fetal_T3 levels. The SSC4 QTL had one significant 1-Mb windows for piglet_T3; the SNP with highest PPI in this window was located at 109681421bp. The SSC4 QTL had two consecutive significant 1-Mb windows for fetal_T3; the SNPs with highest PPI in this window was located at 112497789bp and 1,13519433bp. The WUR SNP was not found to be significantly associated with thyroid hormone levels in the GWAS of the current study. However, the co-localized QTL on SSC4 for piglet_T3 and fetal_T3 may be a good target to improve both respiratory and reproductive PRRS.

A QTL for fetal_T4 levels that explained 6% of the GV was identified on SSC13 between 29 and 31 Mb. Previous works have identified 68 nearby QTL in this region including those for immune-related traits such as leukocyte cell subset percentages (Lu et al., 2011), susceptibility to the pig respiratory pathogen *Actinobacillus pleuropneumoniae* (Reiner et al., 2014), and susceptibility to *Salmonella* (Galina-Pantoja et al., 2009). In addition, there are reports of a QTL for the reproductive traits number of stillborn pigs (Onteru et al., 2012) and corpus luteum number (Bidanel et al., 2008; Hernandez et al., 2014). The corpus luteum is essential for the establishment and maintenance of pregnancy and primarily functions in the secretion of the hormone progesterone (Niswender et al., 2000). Moreover, a previous study in pigs found that thyroid hormones augment the effect of follicle stimulating hormone on cultured granulosa cells (Maruo et al., 1987). If the region on SSC13 identified in the current study altered maternal thyroid levels as well, the two factors could be related. Progesterone therapy has been shown to increase T4 levels in humans (Sathi et al., 2013) and has been shown in swine to impact the thyroid gland (Sekulić et al., 2007).

Another QTL for fetal_T4 levels that explained 3% of the GV was identified on SSC15. This QTL had two consecutive significant 1-Mb windows for fetal_T4; the SNPs with highest PPI in this window were located at 117674402bp and 120106066bp. Previously identified nearby QTL for immune-related traits in this region include blood levels of the interleukin-8 (IL-8) cytokine (Dauben et al., 2021), leukocyte cell subset percentages (Lu et al., 2011; Wang et al., 2013) and susceptibility to *Salmonella* (Galina-Pantoja et al., 2009). Four previously identified QTL were identified for this region for corpus luteum number (Rohrer et al., 1999; Hernandez et al., 2014; Schneider et al., 2014), with implications discussed above. In addition, an association has been reported in this region for birth weight variability (Wang et al., 2016). Thus, these are interesting SSC15 co-localizations due to the well-known impacts of thyroid hormone on weight and the high genetic correlation between fetal_T4 and BW reported in the current study.

### 4.4 Putative candidate genes identified that may be associated with host resilience and susceptibility

All annotated positional candidate genes were identified for each trait within ±200 Kb for all significant SNP (Supplementary Table S3), as well as for the top three SNPs with the highest PPI within 1-Mb windows that explained the largest amount of GV for each trait (Table 5). The genes involved in producing and regulating thyroid hormones are complex and highly regulated and have been previously reviewed (Gereben et al., 2008; Williams, 2008; Cheng et al., 2010). In addition to the protein coding genes, miRNAs may play a role in regulating thyroid hormone levels (Ali et al., 2021). There are also substantial interactions with other signaling pathways such as neurological development (Bernal, 2007) and metabolic regulation (Liu and Brent, 2010). Because our study investigated the genetic control of thyroid hormones during infection, we anticipated identifying genes involved in host resilience. Here we discuss putative candidate genes by function, with a special emphasis on immune-related genes, and then discuss solute carriers, growth factors, and transcriptional regulators.

In the current study we identified several immune-related genes near QTL for thyroid hormone levels. Links have been established between thyroid hormones and innate immunity (Montesinos and Pellizas, 2019) and adaptive immunity (Rubingh et al., 2020). Thyroid hormones seem to be particularly important in regulating the function of dendritic cells, macrophages, natural killer cells, and neutrophils (van der Spek et al., 2021) with increased levels of thyroid hormones resulting in a proinflammatory response of these cells (Rubingh et al., 2020). These innate immune cells are one of the first lines of defense against PRRSV infection and also function to bridge innate and adaptive immunity (Crisci et al., 2019). Importantly, PRRSV is restricted to replicating in cells of the monocyte lineage expressing sialoadhesion (CD169) and CD163 (Meulenberg, 2000; Whitworth et al., 2015; Burkard et al., 2017) including macrophages and dendritic cells. In the current study we identified *IRF8* (interferon regulatory factor 8) as a putative candidate gene nearby QTL for fetal_T4 (SSC6, 2853231bp). The IRF8 protein is a transcription factor that regulates differentiation of myeloid progenitor cells into monocyte precursor cells (Tamura et al., 2015). *IRF8* also regulates expression of genes stimulated by IFN-alpha and IFN-beta that are typically induced during viral infection (Tailor et al., 2007), and the latter is significantly upregulated in fetal thymus and spleen following PRRSV infection (Pasternak et al., 2020a). This gene should be investigated further to understand the complex interaction of thyroid hormone suppression during PRRSV challenge and macrophage populations. We found candidate genes related to adaptive immunity as well: *CD247* near a QTL for fetal_T3 and *MAPK8* near a QTL for piglet_T3 (SSC14, 89227581bp). The CD247 protein is T-cell receptor zeta that has an important role in antigen recognition and signal transduction and found to be differentially expressed during PRRSV infection (Liang et al., 2017). The MAPK8 protein is a transcription factor that belongs to the well-characterized MAP kinases family. This group of proteins have pleotropic functions involved in cell proliferation, differentiation, and transcriptional regulation of numerous pathways (Zhang and Liu, 2002). *MAPK8* has recently been shown to be differentially expressed in the tonsils of PRRSV infected PHGC pigs (Dong et al., 2021).

Thyroid hormones have been shown to depend on solute carrier proteins (He et al., 2009). We identified five solute carrier genes near QTL including *SLC16A4*, *SLC18A3*, *SLC25A20*, *SLC26A6*, and *SLC39A7*. The most well-studied solute carriers that act as thyroid hormone transporters include SLC16A2, SLC16A10, and SLCO1C1 (Arjona et al., 2011; Müller et al., 2014), and many other transporters have been shown to interact with thyroid hormones to various degrees as reviewed previously (Visser et al., 2011). Several other solute carriers have been implicated in thyroid hormone synthesis (Francis, 2021; Tanimura et al., 2021). Although some of the candidate genes identified here are within the same families as those previously reported to be associated with thyroid hormone levels, we identified novel gene associations with thyroid hormone levels. These solute carrier genes should be investigated further to uncover possible interactions with thyroid hormones. PRRSV infection in the fetus has recently been shown to alter expression of tight junction proteins at the maternal fetal interface (Guidoni et al., 2022). Recent evidence suggests complex interactions between tight junction proteins and cellular adhesion proteins (Campbell et al., 2017); two cadherin genes (i.e., *CDH19* and *CELSR3*) were identified in the current study as candidate genes near fetal_T4 QTLs (SSC1, 155713656bp). In addition, we found transcriptional regulator genes near QTL (SSC4, 83435243bp) including *CREG1*, and *POU2F1*. Gene expression is regulated by thyroid hormones *via* interactions with thyroid hormone receptors, which are DNA-binding transcription factors (Wu and Koenig, 2000). In the current study we identified the *HSDL2* (hydroxysteroid dehydrogenase like 2) gene near a QTL for fetal_T3 levels (SSC1, 252973691bp). In humans HSDL2 modulates cervical cancer cell proliferation and metastasis (Yang et al., 2021). Recent work has shown PRRSV infection in fetus to result in multi-organ cell cycle suppression (Mulligan et al., 2022). In addition, the CREG1 and POU2F1 proteins function in cell proliferation and differentiation pathways and were identified near QTL for fetal_T3 levels in the current study. Finally, we identified *PTH1R* (parathyroid hormone 1 receptor) as well as three growth factor and cellular proliferation genes; *BMP5*, *CSPG5*, and *PROK1*. It has been shown that PRRSV infection in the fetus results in changes in cellular proliferation and angiogenesis at the maternal fetal interface (Barrera-Zarate et al., 2022). The *PTH1R* gene has been shown to be expressed in the thyroid gland and in one study human carriers of a mutation in this gene exhibited hypothyroidism (Calvete et al., 2017). The identification of growth factor-related genes is unsurprising given the high genetic correlation between thyroid hormones and BW measurements identified in the current study.

Overall, a variety of putative candidate genes were identified nearby QTL with interesting biological connections especially the immune-related genes. The genes identified here are good candidates for future investigations into the complex interaction of thyroid hormones and animal health.

## 5 Conclusion

For the first time, we report genomic control of thyroid hormone levels in both piglets and fetuses during PRRSV infection. Our main findings were that thyroid hormone levels following PRRSV infection were heritable and had positive genetic correlations with growth rate. Multiple QTL with moderate effects were identified for T3 and T4 levels during challenge with PRRSV; candidate genes were identified, including several immune-related genes. These results advance our understanding of both piglet and fetal response to PRRSV infection by characterizing the genomic control of a novel phenotype associated with host resilience and susceptibility. Future work should continue to probe the complex interaction of thyroid hormones and immunity.

## Data Availability

The data presented in the study are deposited in the Ag Data Commons repository, doi accession number: https://doi.org/10.15482/USDA.ADC/1528496, as well as the PigQTLdb repository, stable URL: https://www.animalgenome.org/QTLdb/supp/?t=DkOq8P2XuT. All data generated and/or analyzed in this study are also stored in the proprietary PHGC relational database (https://www.animalgenome.org/lunney/) or can be requested by contacting Dr. John Harding (john.harding@usask.ca). The data were generated on samples from commercially owned animals, but access can be provided upon reasonable request to the authors.

## Glossary

BW: Body weight
DPI: Days post inoculation
DPMI: Days post maternal inoculation
Fetal_T3: Triiodothyronine (T3) levels measured in fetal serum at 12 or 21 DPMI, ng/dL
Fetal_T4: Thyroxine (T4) levels measured in fetal serum at 12 or 21 DPMI, µg/dL
GV: Total genetic variation
GWAS: Genome-wide association study
HPT: Hypothalamic-pituitary-thyroid axis
IRF8: Interferon regulatory factor 8
IUGR: Intrauterine growth restriction
JWAS: Julia for Whole-genome Analysis Software
LD: Linkage disequilibrium
LPS: Lipopolysaccharide
LS: Least square
MCMC: Markov chain Monte Carlo
NTIS: Non-thyroidal illness syndrome
PGM: Pregnant gilt model
PHGC: Porcine reproductive and respiratory syndrome host genetics consortium
Piglet_T3: Triiodothyronine (T3) levels were measured in serum at 11 DPI, ng/dL
PPI: Post probability of inclusion
PRRS: Porcine reproductive and respiratory syndrome
PRRSV: Porcine reproductive and respiratory syndrome virus
PRRSV2: Porcine reproductive and respiratory syndrome virus type 2
PTH1R: Parathyroid hormone 1 receptor
QTL: Quantitative trait loci
**r** ^**2**^: Measure of linkage disequilibrium
**r**_**g**_: Genetic correlation
rT3: reverse triiodothyronine
SSC: *Sus scrofa* chromosome
SNP: Single nucleotide polymorphism
T2: Thyroid hormone diiodothyronine
T3: Thyroid hormone triiodothyronine
T4: Thyroid hormone thyroxin
TSH: Thyroid stimulating hormone
VL: Viral load
WG: Weight gain
WG21: Weight gain from 0 to 21 DPI, kg
WG42: Weight gain from 0 to 42 DPI, kg
WUR: SNP WUR10000125 associated with PRRS tolerance near the GBP5 gene
